# Competitive Food and Beverage Policies and Obesity among Middle School Students: Variability by Urbanicity in California

**DOI:** 10.1089/chi.2021.0025

**Published:** 2021-12-23

**Authors:** Mika Matsuzaki, Brisa N. Sánchez, Maria Elena Acosta, Emma V. Sanchez-Vaznaugh

**Affiliations:** ^1^ Department of International Health, Johns Hopkins Bloomberg School of Public Health, Baltimore, MD, USA.; ^2^ Department of Biostatistics, Drexel University, Philadelphia, PA, USA.; ^3^ Department of Public Health, San Francisco State University, San Francisco, CA, USA.

**Keywords:** middle school, obesity, policy, rural, school nutrition, second city, suburban, urban

## Abstract

***Introduction:*** This study examined the association between California school nutrition policies and population-level trends in childhood overweight/obesity by levels of urbanicity.

***Methods:*** We used interrupted time series with Fitnessgram data on overweight/obesity from the period 2002 to 2010 pertaining to African American, Latino, Asian, and White students in seventh grades who attended California public schools. We used multilevel logistic regression models to examine the impact of the introduction of successive California school nutrition policies on overweight/obesity prevalence, stratified by gender and adjusted for school district-, school-, and student-level characteristics.

***Results:*** At the start of the study period, rural areas and second cities (*i.e.,* population centers with lower population densities than urban areas) had relatively low prevalence, but experienced sharp increases in 2002–2004, leading to higher prevalence of overweight/obesity than suburban areas. There was evidence of beneficial policy influences on overweight/obesity in most areas, except for girls in urban areas and boys in second cities. The evidence of beneficial changes was strongest among children attending schools located in rural areas, and boys in suburban and urban areas. These results persisted even after we accounted for differences in racial/ethnic compositions, socioeconomic characteristics of the schools and school neighborhoods, and school sizes, as well as child-level race/ethnicity, age, and student fitness levels.

***Conclusion:*** Despite evidence of beneficial policy impact, childhood obesity prevalence remains high, especially in urban areas in California. Additional policies and environmental interventions are recommended to address obesogenic risk factors unique to each area.

## Introduction

Childhood obesity is a major public health issue across the world. Obesity in childhood is associated with poor health outcomes in later life such as diabetes, cancer, hypertension, and atherosclerosis, as well as premature mortality.^1–3^ In the United States, over 35% of adolescents 12–19 years of age were classified as overweight or obese in 2015–2016, with obesity levels varying across geographic locations and urbanicity.^4^

Policies to regulate “competitive” foods and beverages (CF&B)—items sold separately from thus “competing” with federally supported school meals^5–7^—intend to improve the nutritional content of CF&B and limit student access to unhealthy foods and beverages in schools. Multiple national, state-level, and local studies have examined the associations between these policies and weight status, with varying results.^7–9^ A smaller body of research has noted that the effectiveness of these policies in lowering overweight/obesity differed across gender, grade levels, school neighborhood socioeconomic status, or racial/ethnic groups.^10–17^

Beyond individual characteristics, it is important to investigate how differences in place-level characteristics may interact with the food policies. Previous studies have shown differences in the availability of CF&B and in the presence of policies for promoting healthy lifestyles such as diet and physical activity by urbanicity.^18–21^ There may also be lower availability of policies that support healthy eating strategies (*e.g.*, banning food marketing and promoting fruit and vegetable consumption) in rural/town schools than urban and suburban schools.^21^ Differences in the levels of financial and human resource availability may also influence the schools' ability to adhere to new school nutrition policies. Closer examination of area-level modifiers of the influence of statewide nutrition policies can help inform future strategies for obesity prevention based on localities.

California was among the first states to introduce CF&B policies in schools, starting in 2004. Using statewide student-level data from California public schools, we investigated whether the influence of CF&B policies on overweight/obesity differed by levels of urbanicity among students in the seventh grade. We hypothesized that the policy effects would be strongest in rural areas. The findings from this study highlight the areas in which the influences of the school nutrition policies may have been weaker and where the obesity prevalence remains high.

## Methods

We used an interrupted time series (ITS) approach to evaluate the effectiveness of the California school nutrition policies by urbanicity of school locations. In middle schools, starting on July 1, 2004, Senate Bill (SB) 677 banned the sale of sugary beverages, followed by California SB 965, effective on January 1, 2006, and California SB 12, effective on July 1, 2007, which updated those standards to include further restrictions on beverages (*e.g.*, limiting milk fat content) and snacks (*e.g.*, limiting calories).

### Sources of Data and Study Variables

#### Data sources

Each year from February through March, public schools in California conduct physical fitness test battery (Fitnessgram) and obtain annual measurements of fitness as well as height and weight for public school students in fifth, seventh, and ninth grades. High validity and reliability have been shown for the BMI data collected in schools vs. data collected by trained specialists.^22,23^ Students engage in as much of the entire physical fitness test as they are able to.^24^ Age, gender, race/ethnicity, and grade information are also provided. Fitnessgram data from 2002 to 2010 were merged with information from geocoded addresses publicly available from the California Department of Education's (CDE) databases plus 2000 and 2010 Censuses. The urbanicity information was obtained from Nielsen company's proprietary PRIZM segmentation data.^25^

#### Student-level variables

Child-level overweight/obesity status was calculated from measured height and weight using an established approach; BMI was calculated as weight in kilograms divided by height in meters squared and converted to a BMI*z* score, based on the CDC 2000 growth charts.^26^ Students were classified as “overweight or obese” if their age- and gender-specific BMI*z* scores were at or above the 85th percentile of the reference distribution.^26^ Other student-level variables included gender, age (modeled in years), race/ethnicity (classified as White, Latino, Black, and Asian), and fitness level (classified as meeting or exceeding fitness standards vs. not, based on the Cooper Institute's guidelines for the time to run 1 mile).^27^ Pacer data were used to generate fitness classifications for those students missing mile run data. Students with missing values for any of the variables included in the adjusted models were excluded from all analyses.

#### Urbanicity data

Nielsen defined urbanicity of census tracts based on population density and employment centers and generated four classifications: urban (census tracts located in areas that contain high density neighborhoods and tend to be employment centers, and may expand into densely populated areas outside the city limits); suburban (those moderately densely populated, and while they are connected to urban areas, they are not themselves population centers); second cities (moderately densely populated, like suburbs, but differ in that they are the primary population center of the surrounding areas), and rural (those that have the lowest population density and are located outside the outer suburban reaches of cities).

#### School-level variables

The models were adjusted for several school-level variables because the implementation of the policies may be influenced by school characteristics: total numbers of enrolled students, racial/ethnic student majority, and the percent of students eligible for free or reduced price meals (FRPM). FRPM was used as a proxy for children's socioeconomic advantage since Fitnessgram does not include individual-level socioeconomic information and FRPM is associated with overweight.^28^ School-level majority racial/ethnic student enrollment was based on CDE's data on the percentage of students in four major racial/ethnic groups (White, Black, Latino, or Asian) at each school. Schools were classified as “majority” for a specific racial/ethnic group if 50% or more of the students reported to be in one of these four groups. If no single racial/ethnic group comprised at least 50% of students or the majority of students were from a racial/ethnic group other than White, Black, Latino or Asian, the school was classified as “other or no majority.”

#### School neighborhood socioeconomic variables

To classify each school according to the levels of socioeconomic resources, census data for its surrounding neighborhood were used: neighborhood income (defined and measured as annual median household income of the residents within the school's census tracts); and residents' educational attainment (measured as the proportion of residents ages 25 and older, who completed 16 or more years of education within school's census tracts).

#### School district-level variable

As an additional socioeconomic indicator, the district-level percentage of students eligible for FRPM was included and categorized into quartiles.

### Study Population

In 2002–2010, the Fitnessgram dataset received by the authors included 3,402,694 child-level records for White, Black, Latino, or Asian children in the seventh grade. After excluding missing data on variables included in the models (3.8%), 3,272,748 records, nested in 3017 schools, were kept for the analyses. The study received approval by the California Committee for the Protection of Human Subjects and was exempted from review by the authors' academic institutions.

### Statistical Analyses

The distribution of student- and school-level characteristics was estimated, overall and by urbanicity, using means and standard deviations or frequencies as appropriate.

#### Models

Multilevel logistic regression models were fitted, with child-level overweight/obesity as the dichotomous outcome, to estimate the obesity effect of the school nutrition policies and investigate the modifying effect of urbanicity. The influence of state CF&B policies likely accrues gradually over time; thus, the models estimated the population-level annual changes in overweight/obesity prevalence in the periods before and after the policies. The policy effects were evaluated as the difference in the trend comparing the period after to the period before the policies took effect. This modeling approach fits within the larger framework of an ITS design, one of the strongest designs to evaluate nonrandomized policy interventions.^29,30^

To evaluate if the policies had differential impact on overweight/obesity by urbanicity levels, the models included two terms, one for year since 2002 to capture the slope before the policies and a linear spline term with a knot placed in 2005 that estimated changes in slopes after such policies took effect and the interaction between each of these terms and urbanicity classifications. The models were adjusted for student characteristics, and for time-varying school/school neighborhood- and district-level covariates. Random intercepts and slopes (for year since 2002 and the spline terms) at the levels of districts and schools within districts were included in the models to account for similarity among students and for the possibility of heterogeneity^4^ of trends before and after the policies took effect at the school and school district levels. Random effects were assumed to have a multivariate normal distribution with unstructured covariance matrix at both levels. Separate models were fitted for boys and girls because of the well-documented sex differences in growth and adiposity.^26^

The model coefficients for the time and spline terms and their interactions with urbanicity were combined to obtain estimates and 95% confidence intervals (CI) of the population-level trend before and after the policies for each urbanicity and sex group. The model results are included in Supplementary Table S1. The resulting trends or slopes are interpreted as the annual change in the log odds of overweight/obesity. In addition, the models were used to estimate the prevalence of overweight/obesity for each year of the study period after adjustment for covariates, using 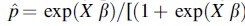 These prevalence estimates were then compared to enable net differences in prevalence from 2002 to 2004 (change over 3 academic years in the prepolicy period) and from 2005 to 2010 (5 years in the postpolicy period). Analyses were conducted in 2019–2020 in R 3.5.1.

## Results

Forty-six percent of the seventh graders in this study attended schools in urban areas (Table 1). Latino students constituted the largest racial/ethnic subgroup in urban areas and second cities, whereas White students were the largest subgroup in suburban and rural areas. The percentages of Black and Asian students were consistent with California demographics, although smaller in rural areas. The crude prevalence of overweight/obesity was highest among students in urban areas and lowest in suburban areas (Table 1). At the start of the study period, rural areas and second cities had relatively low prevalence, but experienced sharp increases since 2002, leading to higher prevalence than suburban areas, which experienced smaller increases. These patterns generally held for each gender separately, although there were some differences in the patterns of crude overweight/obesity trends by sex and urbanicity (Supplementary Fig. S1).

**Table 1.** Characteristics of California Public School Students in Seventh Grade by Levels of Urbanicity, 2002 to 2010

Characteristics	Urban	Second city	Suburban	Rural
Total, *n* (%^a^)	1,493,368 (45.6)	643,759 (19.7)	769,510 (23.5)	366,111 (11.2)
	% or mean	% or mean	% or mean	% or mean
Age				
11	0.2	0.2	0.2	0.2
12	55.4	52.8	54.2	50.4
13	41.4	44	43.2	45.5
14	3	3	2.4	3.8
15	0.1	0.1	0.0	0.1
Gender				
Boys	49.2	49.2	49.1	49.0
Girls	50.8	50.8	50.9	51.0
Race/ethnicity				
Black	9.7	7.3	7.4	3.3
Asian	11.7	5.6	11.1	2.9
Latino	59.9	44.3	38.5	42.7
White	18.8	42.8	43.1	51.2
Overweight/obese^b^				
2002	39.4	33.9	32.2	33.0
2003	40.2	35.7	32.9	35.2
2004	40.6	35.6	33.7	36.2
2005	41.6	37.2	34.1	37.2
2006	41.7	36.4	33.1	36.5
2007	41.4	37.2	33.1	36.3
2008	41.7	36.8	33.1	36.3
2009	41.2	36.7	33.2	36.6
2010	41.2	36.6	33.0	36.7
Physical fitness^c^				
Needs improvement	37.8	32.3	30.4	31.2
Meets standards	45.7	47.3	47.3	46.4
Exceeds standards	16.4	20.4	22.3	22.4

Authors' analyses of the California Fitnessgram data.

^a^ The % by urbanicity in this row is based on the total population. For other percentages in table were calculated within each urbanicity subgroup.

^b^ Overweight and obese categories are defined as having BMI *z*-score ≥85th and 95th percentiles in comparison to the CDC growth chart from 2000 and calculated using raw data on height and weight measures provided by the California Department of Education.

^c^ Those categories are based on whether students exceeded, met, or did not meet the Cooper Institute's guidelines for the time to run a mile for each age and gender.

In suburban areas, school-neighborhoods had greater socioeconomic resources than those in urban areas, second cities, and rural areas. In urban and suburban areas, schools had higher medians for the numbers of enrolled students than second cities or rural areas, whereas those in rural areas had the lowest (Table 2).

**Table 2.** School-Level Characteristics by Urbanicity (2002–2010)^a^

Characteristics	Urban	Second city	Suburban	Rural
	Median	Median	Median	Median
Free or reduced price meal program (%)				
2002	69.8	55.2	38.8	57.6
2005	69.2	52.7	37.2	54.0
2010	71.8	54.1	38.9	53.1
School's neighborhood-level education (%)^b^				
2002	20.4	17.1	30.5	15.8
2005	21.1	17.9	30.3	16.8
2010	20.6	19.1	31.6	18.2
School's neighborhood-level median household income (US dollars)				
2002	50,160	46,507	65,139	39,792
2005	51,631	49,607	69,333	43,152
2010	54,055	52,988	75,962	50,134
Mean school enrollment (*n*)^c^				
2002	873	826	853	271
2005	859	773	843	267
2010	795	731	805	272

^a^ Authors' analyses of school characteristics databases, available publicly within the California Department of Education's website.

^b^ Defined as percentage of residents who have a bachelor's degree.

^c^ Mean of yearly enrollment data across all years for each school.

**Figure 1.**
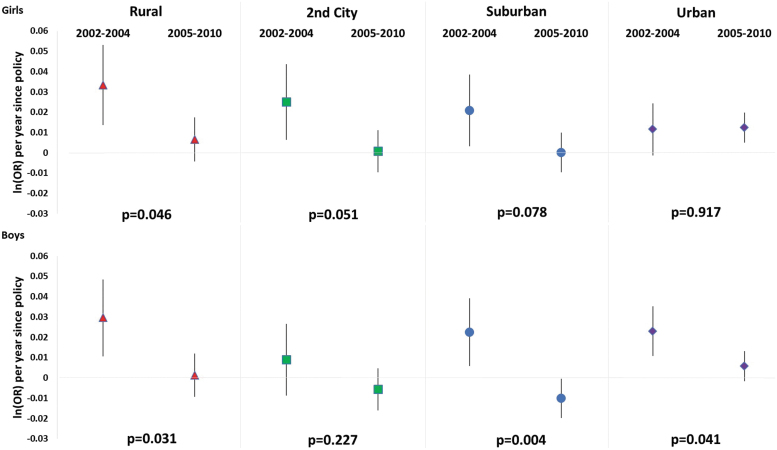
Adjusted log odds of overweight/obesity per year (*i.e.*, trend) within the periods 2002–2004 (baseline, no policies in effect) and 2005–2010 (after California School Nutrition Policies) by urbanicity by gender: seventh grade students. Positive values indicate an increasing population-level trend in overweight/obesity prevalence; negative values indicate a decreasing trend, while values that are not different from zero represent a plateau. The *p*-values are for the test of whether the annual log odds of overweight/obesity changed significantly after the policies took effect, compared to the period before the policies were in place. Color image is available online.

**Figure 2.**
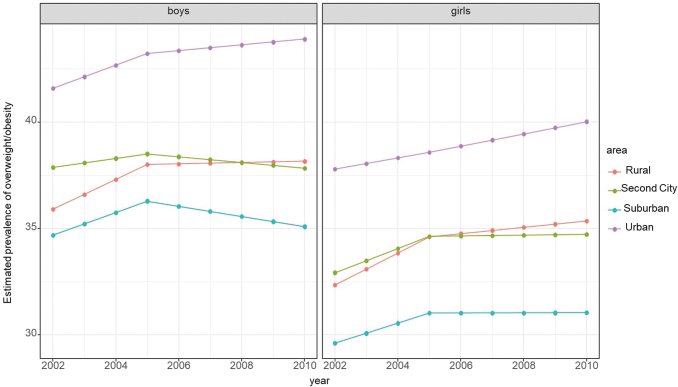
Estimated prevalence of overweight/obesity prevalence among seventh graders by urbanicity of school locations and by gender of students. Overweight/obesity prevalence estimates were derived from a logistic regression model, adjusted for student, school, school neighborhood socioeconomic factors, and district-level percent of students eligible for free or reduced price meals. For each urbanicity and sex strata, trend lines are shifted up or down on the vertical axis so that the prevalence averaged across all years matches the observed overweight/obesity prevalence. These vertical shifts do not impact the slope of the trends. Color image is available online.

Figure 1 shows the estimated annual changes in log odds of overweight/obesity by urbanicity of the school neighborhoods before (2002–2004) and after (2005–2010) the California nutrition policies took effect. Based on the model results, the estimated trends in overweight/obesity prevalence by urbanicity and by gender are displayed in Figure 2. Overall, there was clear evidence of differential influences of the policies by urbanicity among boys (*p* = 0.02), but not for girls (*p* = 0.2). During the baseline period when there were no policies in effect, the overweight/obesity prevalence increased in all subgroups, with the exception of boys in second cities and girls in urban areas. In the period after the California school nutrition policies took place (2005–2010), overweight/obesity significantly decreased among boys in suburban areas and plateaued among all other groups, except girls in urban areas. The evidence of beneficial changes in overweight/obesity trends (slope) comparing the postpolicy to the prepolicy periods was strongest for children in rural areas [girls: *β*: 0.03 (95% CI: 0.01–0.05); boys: 0.03 (0.01–0.05)] and boys in suburban [0.02 (0.01–0.04)] and urban areas [0.02 (0.01–0.04)], and moderate for girls in second cities and suburban areas. The annual log odds of overweight/obesity for girls in urban areas remained at the same magnitude after the policy compared to before [0.00 (−0.01 to 0.01)], although the estimate had higher precision in the postpolicy period. For boys in second cities, there was no clear evidence of prepolicy and postpolicy change.

## Discussion

We observed favorable changes in overweight/obesity prevalence trends following the California school nutrition policies for all urbanicity groups, except for boys in second cities and girls in urban areas. The evidence of beneficial changes in overweight/obesity trends between pre- and post- policy periods was strongest among children attending schools located in rural areas, and boys in suburban and urban areas. While the policy influences did not fully mitigate the increases in prevalence in the prepolicy period—especially the sharp increases in rural areas from 2002 to 2004—the favorable overweight/obesity trends are encouraging. Nevertheless, the overweight/obesity prevalence among children remains high in most areas in California, especially in urban areas.

A prior study found that the school nutrition policies in California were associated with a lower annual increase in overweight/obesity among the seventh graders overall.^14^ Our study builds upon those results by examining whether policy influences on obesity varied in specific geographic locations defined by levels of urbanicity. While studies often dichotomize urbanicity levels—including suburban areas from urban or rural areas^4^—the sizable and diverse data used in this study enabled us to separate out less dense second cities and suburban areas. Second cities, those with lower population density, and suburban areas are distinct from urban and rural areas in terms of environmental and socioeconomic characteristics. Our analysis examined the potential variations in the school policy’ influences on obesity at a more granular level of urbanicity.

We observed promising changes in rural areas, where the overweight/obesity prevalence went from increasing to plateauing trends after the policies went into effect. However, while we observed some evidence of beneficial influences of these policies, the overweight/obesity prevalence remains high in rural areas. Previous research has shown that rural schools may have less healthy food environments than urban or suburban schools,^31^ and local policies governing foods and beverages may be weaker and less available in rural than urban areas.^21,32^ Statewide policies may have been able to improve school food environments in a more equitable manner across geographic locations, but they may be insufficient to reverse the sharp increases in prevalence in rural areas during the prepolicy period. Factors outside school settings may have continued to substantially influence children's weight status in rural areas. Longer distances from home to grocery stores^33^ as well as difficulties in accessing supermarkets may drive parental food purchasing decisions and subsequently influence children's exposure to healthy and unhealthy foods at home.^34–38^ Even when the school food environments improve, these environmental factors unique to rural settings may present barriers to maintaining healthy dietary behaviors outside school settings.

Second, we observed weaker evidence of beneficial changes among boys in urban areas compared to boys in suburban areas and among girls in urban areas in comparison to all other areas. Food environments differ by urbanicity^39^ and food environments near urban schools may interact with students' dietary behaviors, and thus undermine policy effectiveness. Another possibility is that some schools in urban areas may have had better initial food environment within schools for both competitive entrees and reimbursable school meals than rural areas.^40^ A longitudinal study showed that the increase in convenience stores near school neighborhoods was associated with higher BMI among girls and school children in urban areas, but not among boys or those in nonurban areas.^41^ This may at least partly explain why we did not observe changes in the upward trend in overweight/obesity among girls in urban areas after the policies took effect.

Third, our analyses showed evidence of declining overweight/obesity trends associated with the policies among boys in suburban areas, highlighting the importance of analyses disaggregated by place. This pattern among boys may be due to prepolicy food environments within schools, levels of policy implementation and compliance, resource availability, food environments and consumption patterns at home and outside of school and home settings, and peer cultures. A national study observed the availability and purchase of CF&B in schools were higher in suburban than other areas.^42^ Suburban schools in this study on average had greater socioeconomic advantages, suggesting that students at these schools may have greater economic power to purchase CF&B items. In addition, socioeconomically advantaged parents may be more likely to limit children's exposure to unhealthy food at home.^43^ Thus, schools may be one of the few places, especially in suburban areas, where children can access, purchase, and consume CF&B.

Finally, it is important to note that the crude overweight/obesity prevalence data showed consistently higher prevalence over time in urban areas and lower prevalence in suburban areas in comparison to other areas in California. The high prevalence in urban areas seen in this study is consistent with a prior cross-sectional study that used California Fitnessgram data in 2010–2011,^44^ but is in contrast to a previous meta-analysis of US studies, which suggested higher childhood obesity in rural than urban areas.^4^ The differences in findings may be partially due to the ways in which urbanicity was defined in each study or regional differences in patterns of association between urbanicity and childhood obesity.

### Implications for Research and Future Policies

While our findings support beneficial impact of the California CF&B policies, additional work is needed to fully mitigate the childhood obesity epidemic. There is a need to strengthen policies to regulate the quality of foods and beverages offered and/or sold to students in schools, particularly among populations that have the highest prevalence of overweight/obesity.^45,46^ Greater availability of multidimensional and cross-sectoral policies and programs in high-risk areas can help build healthier environments for children in communities, neighborhoods, and homes,^47^ and in turn promote healthy weight and increase overall child health. Future research should examine context-specific mechanisms that underlie the potential differences in policy effects across subgroups and geographic locations.

### Limitations

Although this study had the advantage of a large sample size, the school nutrition policies were nonrandomized. Thus, there was no clear comparison group since policies went into effect at the same time in California public schools. The use of an interrupted time series design is one of the strongest designs to evaluate nonrandomized policy interventions; however, we cannot unequivocally infer that the favorable changes we observed were solely attributable to the policies. We were unable to account for local-level policies that may have been in effect, which may explain the observed findings; however, such policies and programs would need to have coincided with the timing of the policies examined in this study. Moreover, information on the implementation and compliance by urbanicity was unavailable for the policies examined in this study, although previous research found that, in California, compliance with CF&B policies was acceptable (67% for foods and 78% for beverages in 2008) and improved over time.^48^ We removed students who had missing data on covariates (3.8%); although given the large size of the data, this is unlikely to materially change the findings..

## Conclusion

To our knowledge, this is one of the first studies to investigate differential influences of the California school nutrition policies on overweight/obesity by the levels of urbanicity. The strengths of evidence and the magnitudes of beneficial influences of these policies varied across urbanicity groups. The evidence of beneficial influences on overweight/obesity trends was strongest in suburban areas, which also had lower prevalence throughout the study period in comparison to other areas. Future studies to elucidate area-specific mechanisms can support the development of additional strategies to mitigate disparities in childhood obesity by locality.

## Supplementary Material

Supplemental data

Supplemental data
