# Supplementary material for: Competitive Food and Beverage Policies and Obesity among Middle School Students: Variability by Urbanicity in California
Source: Child Obes. 2021 Dec 23;18(1):41–9. doi: 10.1089/chi.2021.0025 (PMC8818511; doi:10.1089/chi.2021.0025)
Supplement: Supplemental data [file Suppl_TableS1.docx]

Supplemental Table 1: Model estimated values for the yearly changes in log odds ratio of being overweight/obesity by sex and urbanicity of school locations among 7^th^ graders. OR=odds ratios. CI=confidence intervals.

|  | Girls |  | Boys |  |
| --- | --- | --- | --- | --- |
|  | β (95% CI) | p-value | β (95% CI) | p-value |
| Before the policies (2002-2004) |  |  |  |  |
| Rural | 0.03(0.01 to 0.05) | 0.001 | 0.03(0.01 to 0.05) | 0.002 |
| Second Cities | 0.03(0.01 to 0.04) | 0.008 | 0.01(-0.01 to 0.03) | 0.318 |
| Suburban | 0.02(0 to 0.04) | 0.021 | 0.02(0.01 to 0.04) | 0.008 |
| Urban | 0.01(0 to 0.02) | 0.076 | 0.02(0.01 to 0.04) | <0.001 |
| After the policies (2005-2010) |  |  |  |  |
| Rural | 0.01(0 to 0.02) | 0.259 | 0.00 (-0.01 to 0.01) | 0.805 |
| Second Cities | 0.00(-0.01 to 0.01) | 0.89 | -0.01(-0.02 to 0) | 0.284 |
| Suburban | 0.00 (-0.01 to 0.01) | 0.978 | -0.01(-0.02 to 0) | 0.041 |
| Urban | 0.01(0 to 0.02) | 0.001 | 0.01(0 to 0.01) | 0.12 |
| Changes between periods with and without the policies |  |  |  |  |
| Rural | -0.03(-0.05 to 0) | 0.044 | -0.03(-0.05 to 0) | 0.031 |
| Second Cities | -0.02(-0.05 to 0) | 0.05 | -0.01(-0.04 to 0.01) | 0.228 |
| Suburban | -0.02(-0.04 to 0) | 0.078 | -0.03(-0.05 to -0.01) | 0.004 |
| Urban | 0.00 (-0.02 to 0.02) | 0.927 | -0.02(-0.03 to 0) | 0.04 |
