# Supplementary material for: Competitive Food and Beverage Policies and Obesity among Middle School Students: Variability by Urbanicity in California
Source: Child Obes. 2021 Dec 23;18(1):41–9. doi: 10.1089/chi.2021.0025 (PMC8818511; doi:10.1089/chi.2021.0025)
Supplement: Supplemental data [file Suppl_FigureS1.docx]

**
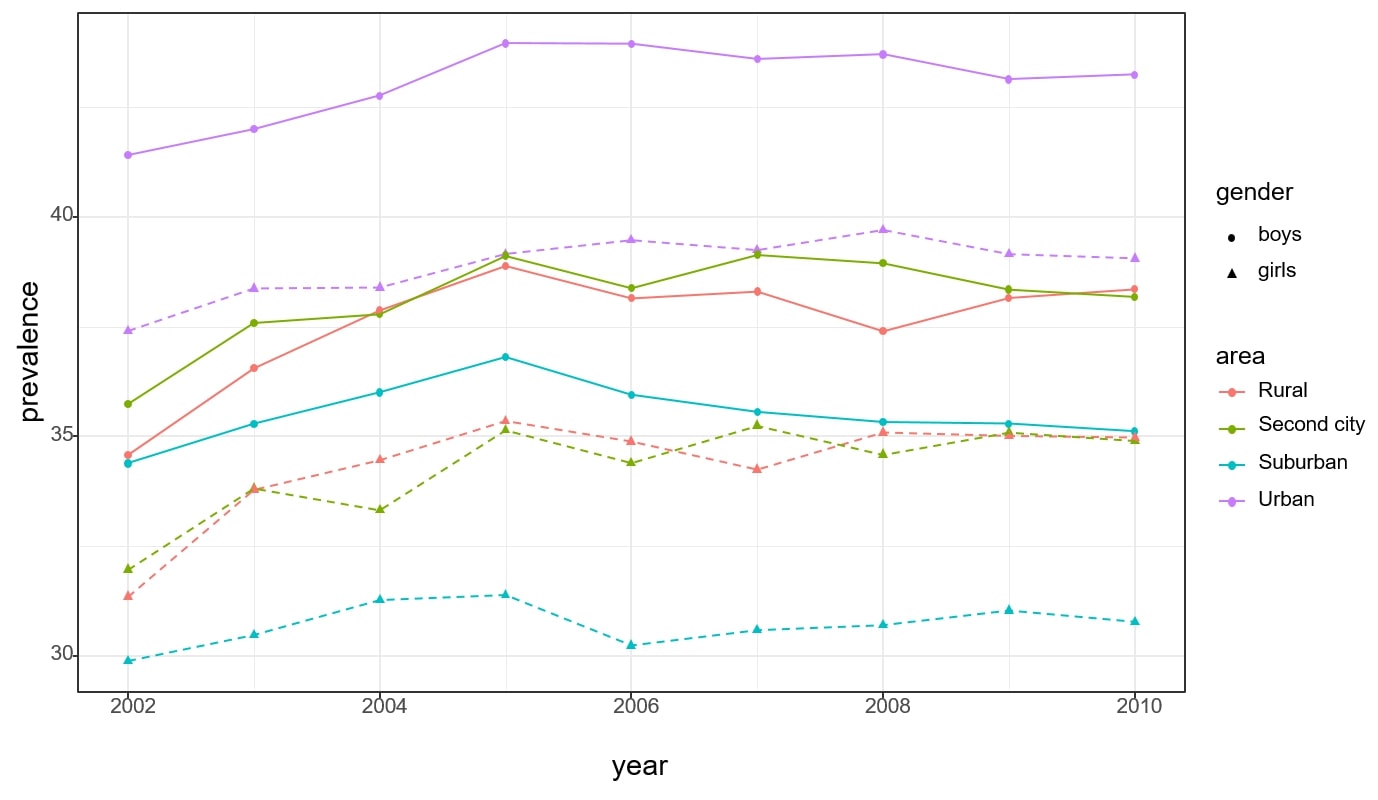
**

**Figure S1: Crude prevalence of overweight/obesity by urbanicity of school neighborhood and by gender among 7^th^ graders in California public schools in 2002-2010.**
